# Evaluation of cytokeratin-19 in breast cancer tissue samples: a comparison of automatic and manual evaluations of scanned tissue microarray cylinders

**DOI:** 10.1186/1475-925X-14-S2-S2

**Published:** 2015-08-13

**Authors:** Cristina Callau, Marylène Lejeune, Anna Korzynska, Marcial García, Gloria Bueno, Ramon Bosch, Joaquín Jaén, Guifré Orero, Teresa Salvadó, Carlos López

**Affiliations:** 1 Molecular Biology and Research Section, Hospital de Tortosa Verge de la Cinta, IISPV, URV, Tortosa, 43500, Spain; 2 Laboratory of Processing Systems of Microscopic Image Information, Nalecz Institute of Biocybernetics and Biomedical Engineering, Polish Academy of Sciences, Warsaw, 02-109, Poland; 3 Department of Pathology, Hospital General Universitario de Ciudad Real, Ciudad Real, 13005, Spain; 4 VISILAB, Engineering School, Universidad de Castilla-La Mancha, Ciudad Real, 13005, Spain; 5 Pathology Department, Hospital de Tortosa Verge de la Cinta, IISPV, URV, Tortosa, 43500, Spain; 6 USR Terres de l'Ebre Institut d'Investigació en Atenció Primària Jordi Gol (IDIAP Jordi Gol), UAB, Tortosa, 43500, Spain

**Keywords:** Tissue array analysis, immunohistochemistry, image processing, algorithms, tumour markers, breast neoplasms

## Abstract

**Background:**

Digital image (DI) analysis avoids visual subjectivity in interpreting immunohistochemical stains and provides more reproducible results. An automated procedure consisting of two variant methods for quantifying the cytokeratin-19 (CK19) marker in breast cancer tissues is presented.

**Methods:**

The first method (A) excludes the holes inside selected CK19 stained areas, and the second (B) includes them. 93 DIs scanned from complete cylinders of tissue microarrays were evaluated visually by two pathologists and by the automated procedures.

**Results and conclusions:**

There was good concordance between the two automated methods, both of which tended to identify a smaller CK19-positive area than did the pathologists. The results obtained with method B were more similar to those of the pathologists; probably because it takes into account the entire positive tumoural area, including the holes. However, the pathologists overestimated the positive area of CK19. Further studies are needed to confirm the utility of this automated procedure in prognostic studies.

## Introduction

In the late 1990s, tissue microarray (TMA) technology began to revolutionize the investigation of potential prognostic and predictive biomarkers [1]. This technology has facilitated high-throughput immunophenotypic analysis in a large series of tissues from different patients on a single glass slide and can serve as a powerful research tool [2].

TMAs can be used to study tissue morphology, protein and gene expression and chromosomal aberrations using different stains, such as those of immunohistochemistry (IHC) and in situ hybridization. The combination of TMAs with clinical samples is an elegant and cost-effective approach to studying panels of biomarkers under identical experimental conditions and to developing prognostic or predictive patterns of patient outcomes [3]. The degree of correlation between TMAs and whole-tissue sections may not be considered ideal at the diagnostic level for individual patients, but is widely regarded as adequate for research purposes [4].

IHC, a cheap and accessible diagnostic technique, is used in daily clinical practice in pathology departments. This technique is essential for the in situ assessment of protein expression, complements morphological information with molecular information, and enables the prediction of responses to targeted therapy [5]. Antibodies used in IHC are the most frequently used in modern biomedical research and the abundance of IHC studies over the last 20 years attests to the technique's popularity [6]. IHC combined with TMA technology increases the throughput of protein expression analysis in tissues and improves assay reproducibility [7,8]. However, the strategy generates a large amount of information that requires painstaking and time-consuming interpretation. The method most commonly used to evaluate and quantify IHC staining in TMAs is visual microscopical analysis, but it is extremely tedious, prone to error and can outweigh the advantages of the high-throughput TMA format. In addition, human interpretations are highly subjective because of the difficulty of establishing the staining intensity parameters, thereby predisposing the process to inter- and intra-observer variability [9,10].

In recent years, pathology procedures have become significantly more automated. Slide preparation, staining, scanning and digital image (DI) analysis of samples have all benefited from such automation. Recent technological advances have made it possible to acquire and store high-quality DIs [11]. Several platforms are commercially available for scanning tissue sections and generating DIs of whole slides. Also, several commercial image analysis applications for IHC quantification are available for some biomarkers and have received clearance from the US Food and Drug Administration (FDA). Digital imaging technology allows the interpretation of IHC results to be standardized, avoiding visual subjectivity and providing more reliable and reproducible results [12,13]. The combination of image analysis software readily available from the public domain, like Image J, with the most commonly used IHC staining methods in surgical pathology practice, is becoming an important approach to diagnostic pathology and research with regard to prognosis and novel targeted therapies for pathologies of the breast and other tissues [14].

Many published studies have compared the results from automated procedures and from visual evaluation of DIs from a small portion of tissue in TMA cylinders [12,15]. Some studies have observed that the variability depends not only on the location of the stain in the cell [16], but also on the number and distribution of the cells [17]. Nevertheless, the variability due to the evaluation of a whole image of each cylinder of the TMA in images obtained by digital scanning of TMA has not been thoroughly investigated. In this study, we present an automated processing procedure with two variant methods developed in Fiji (Image J) for quantifying the IHC marker cytokeratin-19 (CK19) in breast cancer tissues using DIs of TMA cylinders. CK19, the main cytoskeleton protein of epithelial cells, is highly expressed in tumoural breast cancer cells [18,19] and is the most common single marker used for detecting disseminated tumour cells [20]. The results obtained by the two automated methods were compared with those from the visual quantification of the same DIs by two trained pathologists.

## Material and methods

### Tissue microarray preparation and immunohistochemistry

93 samples of ductal invasive breast cancer diagnosed between 2000 and 2007 were selected from the collection of the Tumour Banks of the Pathology Department of the Hospital Verge de la Cinta. Two cores of representative tumour area were selected by an expert pathologist from each paraffin-embedded breast tissue biopsy of the patient. The 2-mm diameter cores were distributed into ready-made holes in a paraffin block using the Arraymold tool. The TMAs contained 50 holes.

For IHC, 3 µm-thick sections of TMAs were dried, deparaffinized in xylene, rehydrated in graded ethanol, and washed in water and PBS [17]. Each slide was immunostained with the monoclonal antibodies directed against the CK19 antigen (CK19; clone RCK108, Dako, Carpinteria, CA). The IHC technique was performed by the ENDVISION^TM ^FLEX (Dako, Carpinteria, CA) method, using the chromogen diaminobenzidine (DAB) as a substrate. Finally, tissues were counterstained with haematoxylin, dehydrated and mounted according to the manufacturer's instructions and laboratory protocol. The entire process was standardised to ensure high reproducibility and stain homogeneity, since these are very important requirements for image analysis [21] and also reduce the costs. The study received approval from the scientific and ethical committee from Hospital Joan XXIII.

### Image acquisition

All stained slides were scanned with the Slide Scanner Aperio ScanScope XT at 40X magnification (20X with 2X magnification changer) to obtain DIs of TMAs. The same white balance values were used during the scanning of the slides to ensure maximum reproducibility between the illuminations of the DIs and to minimize any differences in the automated evaluation of the markers. The final resolution of the captured images was 0.25 µm/pixel [11]. The correct digitization of each TMA was checked using ImageScope software. The mean size of each scanned TMA was around 30 GB. Each cylinder comprising the TMA was then extracted as an individual DI with algorithms developed by the VISILAB group of the University of Castilla-La Mancha, Spain. Each digital image corresponded to one cylinder and was assigned an individual identification number. The DIs obtained were saved in uncompressed tagged-image file (TIFF) format.

### Manual quantification

For visual quantification, each DI was opened in Fiji. Two trained pathologists from the Hospital de Tortosa Verge de la Cinta directly evaluated digital images of each case on a computer screen, determining the percentage of the total area of the cylinder that was positively stained with CK19. Before manual evaluation, evaluation criteria were agreed by the pathologists, since quantification of the percentage of the CK19 positive area had no previously established criteria as part of their daily practice. All results were exported to a Microsoft Excel 2003 worksheet.

### Automated quantification

The automated quantification procedure consisted of two steps, carried out without previous image calibration: the evaluation of the total area of each cylinder and the evaluation of the area of each cylinder that was positively stained with CK19. Images were analysed with Fiji image processing software, which supports a macro language for specific procedures that allows the sequential reproduction and automation of all steps a Lab colour model with L channel in range 0-255.

#### First step: evaluation of total area of cylinder

**Figure 1:**
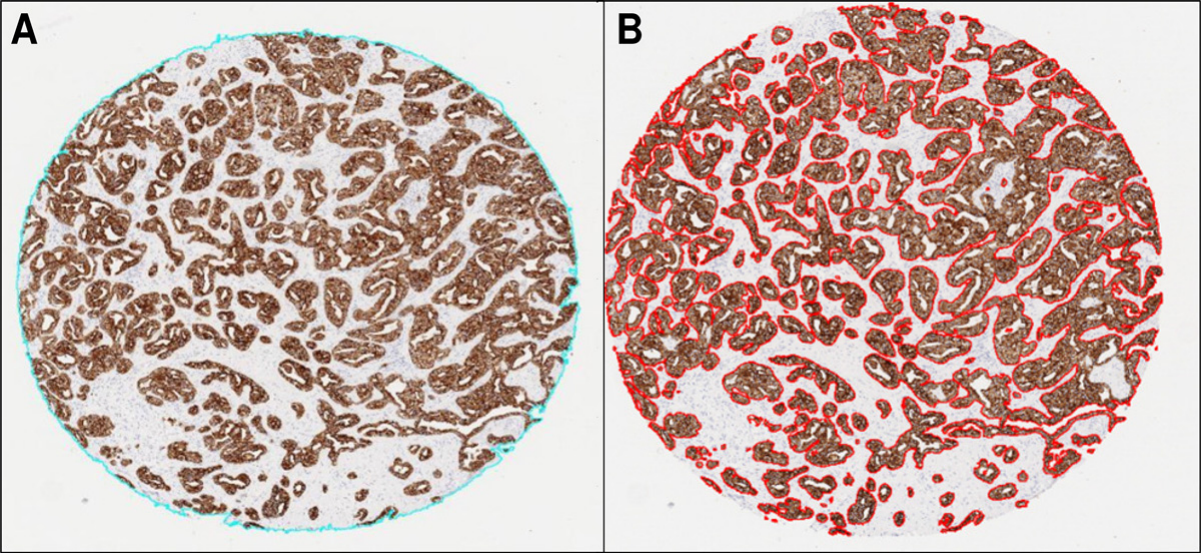
**Steps in the automated quantification procedure**. First step, with the total area of the cylinder delimited in blue (A). Second step, with the area selected inside the TMA cylinder marked in red (B).

In this step, the total area of each cylinder was calculated as the total number of pixels inside the cylinder by using the L channel of CIE L*a*b* colour model and applying a median filter before segmenting the image. First, the digital image was divided into the three greyscale channels of the model colour CIE L*a*b* and the L channel was selected for further processing. This channel contained the lightness image information, allowed better discrimination between the values of the pixels inside and outside the cylinder based on thresholding. A 3x3 median filter was then applied to the L channel image, which replaced each pixel inside the cylinder with the median of neighbouring pixels. This filter reduced noise and homogenised pixel values inside and outside the cylinder. Finally, the pixels inside the cylinder were segmented with the threshold tools of Fiji software in order to select those objects containing pixels with greyscale values from 0 to 238 and with an area larger than 1 million pixels. It was applied in order to select all the pixels that made up the cylinder. Under these conditions we were able to select a single object representing the entire cylinder (Figure 1A).

#### Second step: quantification of CK19-stained area

The second step evaluated the total number of positive brown pixels inside each cylinder. For this purpose, the original image was split into three single images, one for each colour channel of the RGB model. Then, an empirical method to establish a "colour translation" formula to create a "brown channel" was used. The formula, developed by Ruifrok, generated a greyscale image with the brown channel by applying the following mathematical operations to the RGB channels: Brown channel = Blue-0.3*(Red + Green). It is a completely automatic method with no need for intensity or colour calibration, or to know in advance the spectral properties of the pure dyes that are to be separated [22]. Subsequently, the greyscale image was segmented using a threshold from 0 to 70 from range 0-255 to select the brown colour pixels and thereby the tumour area stained by CK19. Finally, after evaluation of different cut-offs, we selected the objects with an area greater than 1000 pixels in order to exclude background pixels and artefacts. The sum of all the segmented areas corresponds to the total positive area of CK19 staining (Figure 1B).

At the end of the second step, two variant methods (A and B) were employed to determine the total tumour area stained with CK19. Method A excluded the segmentation holes inside the positively stained area. These holes are the pixels that correspond to the light of the tumour glands and the pixels of the nuclei inside the segmented objects. In method A, the final result was the sum of all positive brown objects, consisting of all pixels corresponding to the tumour area stained with CK19. Conversely, method B consisted of the same positive objects as in method A and the pixels of the segmentation holes inside the positively stained area. The final result of method B was the sum of all positive brown objects, the nuclei and the light of the tumour glands of these objects.

### Calculation of the positively stained CK19 area

All the values corresponding to the total number of pixels in the cylinder and the pixels of the stained area inside the cylinder were automatically exported to a Microsoft Excel 2003 worksheet. The percentages of positively stained CK19 area were taken as the ratio of the number of brown pixels evaluated in the second step of the procedure (method A or B) to the total number of pixels in each cylinder, as determined in the first step of the procedure.

### Statistical analysis

All statistical analyses were done using SPSS version 21.0 (SPSS Inc., IBM). The intraclass correlation coefficient (ICC), Bland-Altman and Kaplan-Meier analyses, with their corresponding graphical output, were used to evaluate the agreement between the results of the pathologists' observations (inter-observer) and between the results of each pathologist with each automated method. The results of the two automated methods (inter-method) were compared solely with the Bland-Altman graphs.

The Bland-Altman analysis assumes that neither system is a gold standard but merely compares two methods or procedures. The conditional probabilities of observing a difference between paired measurements were estimated by the Kaplan-Meier procedure. The ICC is a measure of the reliability of measurements or ratings, for the purpose of assessing inter-rater reliability. In this study we calculated the ICC of absolute agreement, which included the variability due to the observers. The ICC was calculated from a two-way random-effects analysis of variance with an index of agreement ranging from 0 (no agreement) to 1 (perfect agreement). The following ICC interpretation scale was used as poor (below 0.40), acceptable (0.40-0.74) and excellent (0.75-1) [23].

## Results

### Inter-observer comparisons

**Figure 2:**
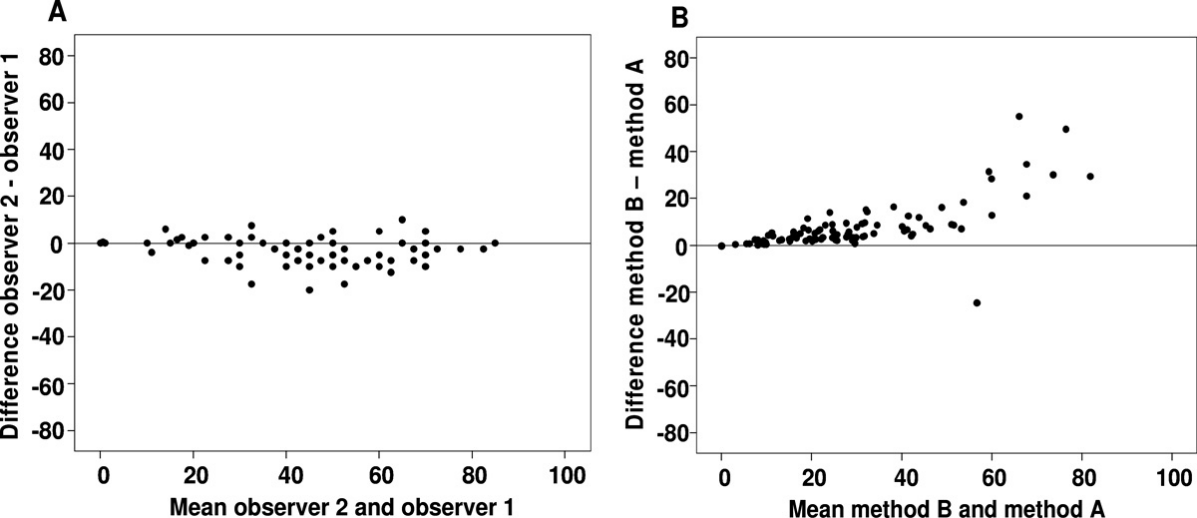
**Overall results obtained by manual and automated evaluation**. Bland-Altman graphs reveal the differences between the two observers (A) and between the two automated methods (B).

Figure 2A illustrates the results of the Bland-Altman analysis, showing the spread of the values around the mean difference between the two observers. These differences were not homogeneous, since observer 1 tended to discern a larger positive area percentage than did observer 2. However, the spread of the differences around the mean difference between the two observers was more or less constant in all digital images and did not appear to be influenced by the complexity of the images (low or high percentages of brown positive objects). These differences ranged from -20% to 10% and 65% of the results had differences of less than 5%. The ICC indicated an excellent level of agreement between the two observers (0.823; 95% CI: 0.631 - 0.905).

### Inter-method comparisons

Figure 2B illustrates the results of the Bland-Altman analysis, showing the spread of the values around the mean difference between the two methods. This demonstrates the close agreement between the two automated methods excluding (method A) or including (method B) the segmentation holes inside the positively stained area, as explained above. The differences between methods A and B were closer to zero when the positive area was less than 10% of the whole; when the percentage positive area was less than 55% the differences between the two methods were less than 10%. On the other hand, when the cylinder area was more than 55% positive, we found differences of 10% to 30% between the two automated methods.

### Comparison between the manual and automated methods

**Figure 3:**
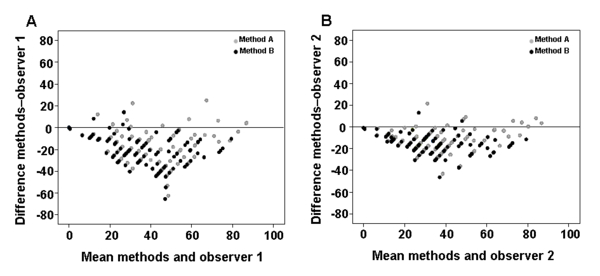
**Superimposed Bland-Altman graphs**. The curves showed the differences between the observer 1 and the two automated methods (A) and between observer 2 and the automated methods (B). Black and grey dots indicate methods A and B, respectively.

Figures 3A and 3B respectively show the superimposed Bland-Altman graphs comparing the results obtained by observers 1 and 2 with those from the two automated methods. The two observers tended to estimate a larger positive area than was calculated by the automated methods. However, observer 1's results showed greater discrepancies with the automated methods than those of observer 2. The differences between the visual and automated methods were more pronounced for method A (holes excluded) than for method B (holes included). The results obtained by observer 2 showed an acceptable level of agreement with those obtained by method A (ICC = 0.663; 95% CI: 0.00 - 0.882) and excellent agreement with those obtained by method B (ICC = 0.772; 95% CI: 0.209 - 0.909). Observer 1's results showed an acceptable level of agreement with those obtained by the method B (ICC = 0.563; 95% CI: 0.001 - 0.794). The ICC between the results of observer 1 and of method A was not an appropriate measure because the condition of equality of variances was not satisfied.

**Figure 4:**
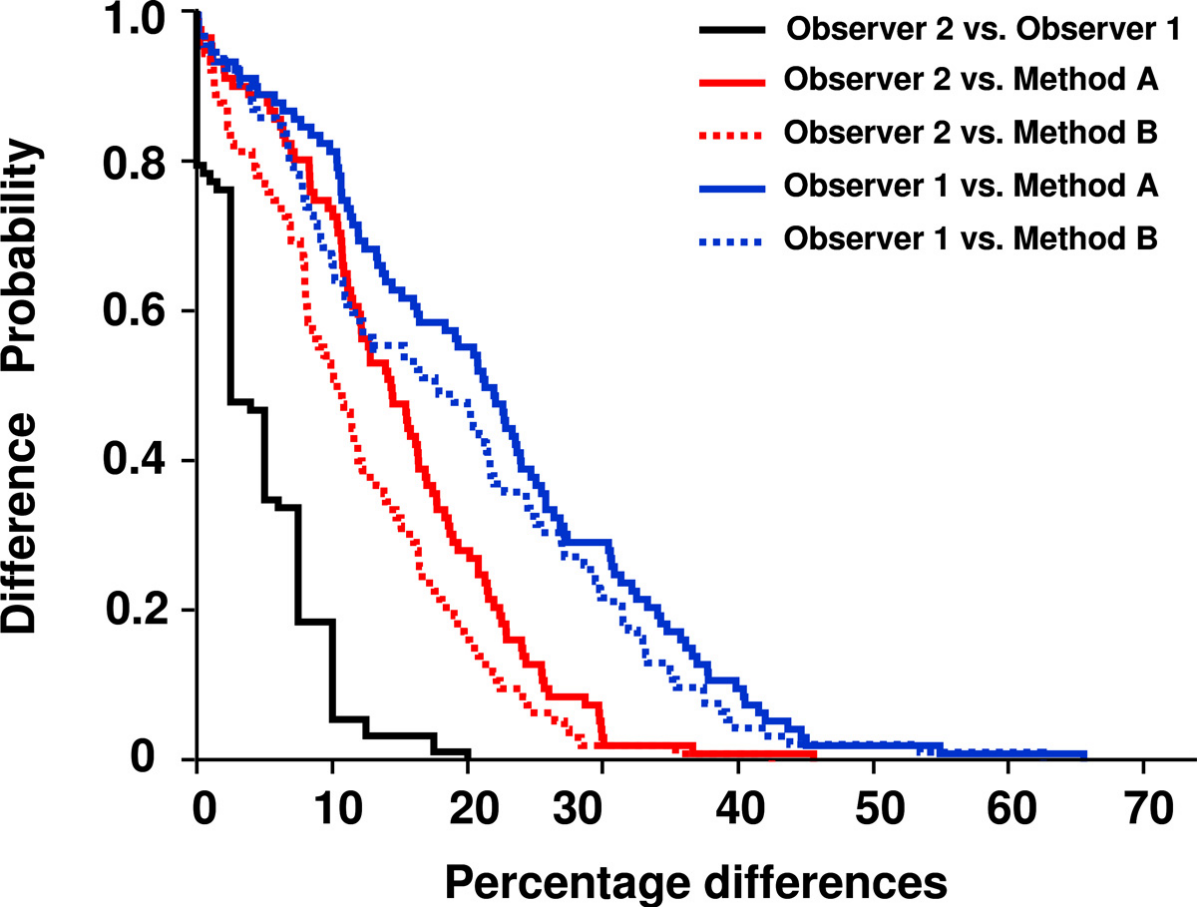
**Superimposed Kaplan-Meier graphs**. The curves compared the probability of difference between the two observers (black line), observer 2 and automated methods A (solid red line) and B (dashed red line), and observer 1 and the automated methods A (solid blue line) and B (dashed blue line).

The conditional probabilities of observing differences between the measurements were estimated by the Kaplan-Meier procedure (Figure 4). Comparison of the Kaplan-Meier curves confirmed the differences between the two observers and between the observers and the two automated methods. The curves indicated that differences were less likely to arise when comparing the two pathologist's counts. The use of method B produced the smallest differences between the results of the automated procedure and those obtained by the two pathologists.

## Discussion

TMAs facilitate high-performance immunohistochemistry, but their analysis presents a number of problems when done manually by optical microscopy. As for single biopsy, the time required, the subjectivity arising from the heterogeneity of staining intensities, and the size, shape and distribution of the cells are major disadvantages of manual evaluation that can be resolved through the use of automated DI analytical procedures. The automated analysis of immunohistochemically stained cells in DIs of complete cylinders extracted from TMAs have been little studied [3,9,24]. Here, we present two automated methods that allow us to measure the percentage of the total area of each cylinder of the TMA that is positively immunohistochemically stained by CK19. We compared these results with the quantifications of two trained pathologists who viewed the same DIs on the computer screen.

The ICC coefficient indicated excellent agreement between the two observers. Nevertheless, the range of differences (-20% to 10%) in the results of the two observers suggested the existence of some inter-observer variability in the evaluation of DIs. Besides the subjectivity of the visual determination of positivity arising from the interpretation of DAB intensity [16], the differences between the observers did not appear to be influenced by the percentage of positive area of CK19. So, to the human eye, the evaluation of the percentage positive area in a large image corresponding to an entire cylinder is not influenced on the cell concentrations. On the contrary, it has been observed that the percentage or the number of positive cells in small images, influence in the variability of human eye evaluation. The difficulties in evaluating the whole area are probably greater because the human eye discerns as positive not only the stained area but also some of the regions within it.

We found a good concordance between the results from automated methods A and B only when the positive area of the cylinders was less than 10%. The larger differences observed when the percentage of positive area in the cylinders was greater than 55% were probably because the number of "tumoural glands" in these DIs is more important. Exclusion (method A) or inclusion (method B) of the pixels corresponding to the light of the tumour glands and the pixels of the nuclei inside the segmented objects, could explain the differences observed in our study. On the other hand, our results also showed that these automated methods tended to identify a smaller positive area than the two manual evaluations. It may be due to the difficulty the human eye has in determining the percentage positive area in large images with a large amount of brown stain without using tools. When manual and automated evaluations of whole slide images of breast tissue stained only with haematoxylin-eosin (without DAB staining) were compared, the two methods proved to have similar accuracy, precision and reproducibility [11]. For both observers, the differences were more pronounced with method A (holes excluded) than with method B (holes included). This may be because the latter method more closely mimics the process of manual quantification. The pathologists interpreted the total positive tumoural area as the area stained with brown colour, the nuclei included in them and also the light of all the tumoural glands. Then, they interpreted the total positive tumoural area, automatically including the total positive tumoural area, rather than solely the brown pixels. However, even when these areas were included in the automated method the pathologists still overestimated the positive area.

Other comparative studies of manual and automated IHC evaluations have already been published [16,25-28]. The pattern of staining of the markers evaluated, the type and size of the DIs analysed, and the manner of portraying the results (e.g., frequency of positive cells, percentage of positive area, and threshold levels of positivity or cut-off values) may explain some of the discrepancies between the manual and automated results obtained in these studies. A relatively good correlation has been found in a study of prostate cancer specimens when the percentage of positively stained areas in a TMA cylinder evaluated by image analysis software was compared to the manual quantification [15]. However, in that study the percentage of stained epithelial cells was reduced to categorical scores (1, <33%; 2 33%-66%; 3, >66%), which simplified the manual evaluation and thereby considerably reduced the differences that may arise between manual and automated methods.

## Conclusion

As compared to manual evaluation, automated image analysis is a simple and economical method of quantifying and scoring immunohistochemically stained markers that improve the levels of sensitivity, precision, reproducibility and standardization of these kinds of measurements. Moreover the advances in automated evaluation of immunohistochemical markers in whole-slide digital images offer a practical means of improving the accuracy and reproducibility of these measurements for diagnosis, education and research purposes. The analysis of complete TMA cylinders provides more information about the prognostic biomarkers in a single image and avoids the loss of information needed to detect prognostic biomarkers. This study is the first part of a project that aims to compare the quantity of different immune response markers in relation to the percentage of the tumoural area (CK19) for the purpose of developing a prognostic factor. Further work is needed to evaluate which of these methods (automated methods A or B versus manual evaluation) will be the best one to use in future prognostic studies.

## List of abbreviations used

TMA: Tissue microarray

DI: Digital image

IHC: Immunohistochemistry

FDA: Food and Drug Administration

CK19: Cytokeratin-19

TIFF: Tagged image file format

DAB: Diaminobenzidine

ICC: Intraclass correlation coefficient

## Competing interests

The authors declare that they have no competing interests.

## Authors' contributions

CC, ML, and CL participated in the conception and the design of the study, images capture and analysis, statistical analysis and drafting of the manuscript. RB and JJ evaluated patient's biopsies and selected representative area for further TMA construction. TS participated in the TMA construction and immunohistochemical staining. AK, MG, GB carried out the standardization of image capture conditions and developing image analysis procedures. GO participated in applying image analysis procedure and database implementation of the results. All authors read and approved the final manuscript.

## Declaration section

Publication of this article was supported by Doctor Ferran Foundation.

This article has been published as part of *BioMedical Engineering OnLine *Volume 14 Supplement 2, 2015: Select articles from the 2nd International Work-Conference on Bioinformatics and Biomedical Engineering (IWBBIO 2014). The full contents of the supplement are available online at http://www.biomedical-engineering-online.com/supplements/14/S2.
